# Effect of concurrent infection of *Helicobacter pylori* with *Toxoplasma gondii* infection on gastric pathology

**DOI:** 10.1186/s12879-024-09270-8

**Published:** 2024-04-16

**Authors:** Marwa A. Gouda, Sara A. Saied, Ahmed Edrees, Rasha Galal Mostafa, Ashraf Elfert, Aya Abdallah seleem, Asmaa Shams, Sameh Afify

**Affiliations:** 1 Clinical and Molecular Parasitology Department, National Liver Institute, Menoufia University, Shibin Elkom, Menoufia, Egypt; 2 Clinical Pathology Department, National Liver Institute, Menoufia University, Shibin Elkom, Menoufia, Egypt; 3 Hepatology and Gastroenterology Department, National Liver Institute, Menoufia University, Shibin Elkom, Menoufia, Egypt; 4 Department of Medical Microbiology, Faculty of Medicine, Menoufia University, Shibin Elkom, Menoufia, Egypt; 5 Clinical Biochemistry and Molecular Diagnostics department, National Liver Institute, Menoufia University, Shibin Elkom, Menoufia, Egypt; 6 Zoonoses department, Faculty of Veterinary medicine, Cairo University, Cairo, Egypt; 7 Department of Pathology, Faculty of Medicine, Menoufia University, Shibin Elkom, Menoufia, Egypt

**Keywords:** Humans, *Toxoplasma*, *Helicobacter pylori*, *Vac As1*, *Cag A*, Histopathological features

## Abstract

*Toxoplasma gondii* (*T. gondii*) and *Helicobacter pylori* (*H. pylori*) are among the most prevalent foodborne parasitic and bacterial infections worldwide. However, the concurrent impact of coinfection on gastric pathology has yet to be studied in depth. The effect of coinfection generally either adds a synergetic or antagonistic impact; we aimed in the current work to assess the impact of *T. gondii* coinfection on the progression of *H. pylori-*associated gastric pathology and reporting *H. pylori* virulent strains. The study was conducted on 82 patients complaining of persistent gastrointestinal symptoms with failed treatment response and prone to endoscopy. They were subjected to stool examination to detect *H. pylori* antigen, serological screening for latent toxoplasmosis, endoscopy, histopathological examination, and molecular detection of *H. pylori* virulence strains in gastric biopsies. Out of the 82 patients, 62 patients were positive for *H. pylori* antigen in stool and 55 patients confirmed positivity by histopathology; out of them, 37 patients had isolated *Vac As1* variants, 11 patients had combined *Vac As1* and *Cag A* variants, and 7 patients had combined *Vac As1*, *Cag A* and *VacAs2* variants. Patients with the combined two or three variances showed significantly deteriorated histopathological features than patients with a single *Vac As1* variant (*P* < 0.05). Latent toxoplasmosis was positive among 35/82 patients. Combined *H. pylori* and *Toxoplasma gondii* infection had significantly marked inflammation than patients with isolated infection (*P* < 0.05). Conclusion: Screening for toxoplasmosis among *H. pylori*-infected patients is recommended as it is considered a potential risk factor for gastric inflammation severity. *H. pylori* gastric inflammation may be heightened by *Toxoplasma* coinfection.

## Introduction

Infectious diseases incorporate a wide range of conditions that significantly threaten the health of humans [26].. Humans are commonly infected with multiple pathogens simultaneously or consecutively, and synergistic and antagonistic pathogenic effects can subsequently impact the overall host responses and the severity of diseases [1].

*Helicobacter pylori* and *Toxoplasma gondii* are among the most prevalent bacterial and parasitic infections, respectively. *H. pylori* is a bacterium characterized by its spiral shape and gram-negative nature, which has a global prevalence, surpassing 50% of the global population, with greater occurrence in developing nations. It is widely recognized as the primary etiological factor responsible for chronic or atrophic gastritis, peptic ulcer, gastric lymphoma, and gastric carcinoma [31]. Considering its carcinogenic properties, the World Health Organization has classified *H. pylori* as a grade I carcinogen [4].

Various virulence factors contribute to the pathogenicity of this bacterium, including *Cag*A, *Vac*A, and others [21]. However, the clinical manifestations associated with the infection are variable [27].

The cytotoxin-associated gene A (*Cag*A) has been recognized as a substantial carcinogen, and *Cag*A-positive strains were associated with an elevated risk of peptic ulcer disease (PUD) or gastric cancer (GC) [18]. The Vacuolating cytotoxin A gene (*Vac* A) is a protein capable of inducing Vacuolation and various cellular activities and is found in all strains of *H. pylori* [29]. It exhibits allelic variation in three primary regions: the signal (s) region (specifically, s1a, s1b, s1c, and s2), the intermediate (i) region (i1 and i2), and the middle (m) region (m1 and m2). The diverse combination of S and M regions plays a crucial role in determining the production of cytotoxic activity and forms a mosaic gene structure [25].

Toxoplasmosis arises from the infection with *T. gondii*, a protozoal parasite capable of invading nucleated cells, including the human brain, eye, and muscle tissue. While human reproduction of cysts was not observed [28], it has been found that muscle and brain cysts can endure throughout the host’s lifespan [14]. The prevalence of *T. gondii* infection is estimated to be 30% globally [9].

The association between toxoplasmosis and other pathogens, either bacterial or viruses has been reported with different outcomes in the form of increased disease severity or protecting effect. The immunomodulatory underlying protozoal coinfection frequently hurts cellular and humoral immune responses toward co-infecting bacterial pathogens. This phenomenon leads to the promotion of bacterial persistence and ultimately results in the manifestation of more severe disease symptoms. Interestingly, the coinfection is believed to cause treatment failure, antibiotic resistance, and inefficient Vaccination programs [1].

One of the reported reciprocal impacts of *Toxoplasma* association with the severity of diseases was the concurrent infection with tuberculosis in an earlier study in Egypt [23]. *Toxoplasma* infection was also positively associated with diabetes type-1 [8]. Studies also reported that schizophrenic patients with *T. gondii* infection have more cognitive impairment [30]. In an earlier study, *T. gondii* infection produced changes in the immunological response to *Helicobacter felis* in experimental mice, making a resistant host susceptible to infection. For both pathogens, there was a notable increase in gastric mucosal levels of IFN-γ and IL-12, but IL-10 levels were dramatically decreased. The alterations were linked to significant stomach lining inflammation, loss of parietal cells, atrophy, and changes in cell structure.There were significant connections between the immune response to different species and indicate that these interactions may affect clinical illness (Stoicov et al., 2004).However, reports on concurrent infection with *H. pylori* and its impact on gastric pathology are lacking. Hence, our study aimed to study the incidence of *Toxoplasma* association among *H. pylori*-infected patients as both pathogens contribute to infection among a large sector of the human population and to document the effect of coinfection on histopathological alteration changes and to report *H. pylori* virulent strains effect on gastric pathology.

## Methods

This cross-sectional study was conducted from January 2023 to June 2023 at the National Liver Institute (NLI) on one hundred patients after approval of the ethical committee under the number N. 00423/2022 from the ethical committee at the NLI. Routine stool examination was done for patients who agreed to participate in the study and complained of persistent gastrointestinal tract symptoms, while individuals with other diseases or having other concurrent intestinal parasites detected by stool examination, individuals under treatment with antibiotics or proton pump inhibitors within the past month, and individuals who were unwilling to participate were excluded. A comprehensive questionnaire was obtained from the patients, encompassing details regarding age, gender, and presenting symptoms.

*H. pylori* surface antigen test: A fecal specimen was obtained from each participant and subsequently preserved at a temperature of -20C for further examination. The detection of *H. pylori* stool antigen (HpSA) was performed using an enzyme immunoassay (EIA) method, following the guidance provided by the manufacturers. Then, it was assessed using the SD Bioline *H. pylori* Ag kit (Standard Diagnostics, Inc.), a commercially accessible product, following the instructions provided by the manufacturer [17].

### Detection of latent toxoplasmosis

The detection of anti-*Toxoplasma* IgG was performed by screening serum samples utilizing commercially available ELISA kits (Abcam, USA). The optical density measurement was conducted at a wavelength of 450 nm, and after that, antibody titer for all samples was determined. According to the manufacturer’s protocol, a positive *Toxoplasma* IgG titer of seven was obtained.

#### Endoscopy

Endoscopy procedures were performed on all patients under the administration of local lignocaine anesthetic. Upper gastrointestinal (GI) endoscopy was conducted using the Olympus X Q40 instrument manufactured by Olympus Optical in Tokyo, Japan. During the endoscopic procedure, two sets of biopsy specimens were obtained from every patient’s antrum and stomach corpus. The initial batch biopsy was submitted for histological analysis and promptly immersed in a 10% formalin solution for fixation. The other specimen was placed into a buffered solution containing 10 mmol/L Tris (pH 8), 10 mmol/L ethylenediaminetetraacetic acid, and 0.5% sodium dodecyl sulfate. It was frozen at -80 °C to facilitate DNA extraction and subsequent polymerase chain reaction (PCR) experiments.

### DNA extraction and polymerase chain reaction (PCR)

The DNA extraction process involved retrieving genomic DNA from stomach biopsy specimens, which was achieved by utilizing the QIAamp DNA Mini Kit (50) 51,304, manufactured by QIAGEN, USA. The extraction procedure was conducted following the directions provided by the manufacturer. A polymerase chain reaction (PCR) was conducted on the extracted DNA using primers to target the *H. pylori* CagA and VacA genes according to Falsafi et al. [11] protocol. The PCR experiment included a negative control sample. The identification of the bands was determined by comparing their diameters with the molecular weight markers of 100 base pairs (Thermo Scientific, (EU) Lithuania). Positive samples were deemed as such when the observable band had a comparable size to that of the positive control DNA. Primers used in the procedures are listed in Table 1.

**Table 1:** List of primers used in this study

Name	Sequence of primer used	Size of detected product	References
*Cag*e A	F-TGCGTGTGTGGCTGTTAGTAG	593 bp	[32]
	R-CCTAGTCGGTAATGGGTTGT		
*Vac* A s1/s2	F-ATGGAAATACAACAAACACAC	259 bp/286 bp	[32]
	R-CTGCTTGAATGCGCCAAAC		

The above-listed primers were used for genotyping *H. pylori* virulent strains. We referred to the product size as base pair (bp)

#### Histopathology

Biopsy specimens were sent to the Pathology Department at the Faculty of Medicine- Menoufia University to create paraffin-embedded tissue blocks. These blocks were then used to produce 4 μm sections. Two sets of tissue slices were made for histopathological analysis. One group was stained with hematoxylin-eosin (H&E), while the other was stained with Giemsa stain (Epredia-USA; Portsmouth, NH, USA), which allowed for identifying *H. pylori*’s effects in the gastric mucosa. The biopsies were evaluated for the extent of inflammatory mononuclear cellular infiltrates {categorized into mild, moderate, or severe}, inflammation activity characterized by neutrophilic infiltrations {either present or absent}, glandular atrophy { present or absent}, metaplasia {present or absent }and atypia { present or absent} [7].

## Results

The study included one hundred patients complaining of recurrent abdominal pain and dyspepsia. First, routine stool analysis was done for all patients with screening for *H. pylori* infection, which revealed that 18 out of the 100 patients had combined parasitic infections (*H. pylori* with either *Entamoeba histolytica* or *Giardia lamblia*) who were excluded from the study. The mean age of the remaining 82 patients was 33.68 ± 6.82 years. Most of the patients were males [68.3%]. Nearly two-thirds of the cases lived in rural areas [61%]. Secondary education and levels below were the prevalent level of education [64.6%]. The most common recorded symptoms were recurrent abdominal pain [100%] followed by dyspepsia [74.4%], then nausea [39%] and recurrent vomiting [14.6] (Table 2).

**Table 2:** Descriptive statistics of socio-demographic data of the studied patients and endoscopic features. General characteristics of the screened group

Gender	Male Female	56 (68.3%) 26 (31.7%)
Age (years)	Mean ± SD (Min-Max)	33.68 ± 6.82 (25–47)
Residence	Urban Rural	32 (39%) 50 (61%)
Level of education	Secondaryandbelow Universityandabove	53 (64.6%) 29 (35.4%)
Clinical manifestations	Recurrent abdominal pain Dyspepsia Nausea Recurrent vomiting	82 (100%) 61 (74.4%) 32 (39%) 12 (14.6%)
Endoscopic Features		
*H. pylori*-positive gastric mucosa (*n* = 55)	Diffuse redness	48 (87.3%)
	Antral nodularity	28 (51%)
	Spotty haemorrhage at the fundus	33 (60%)
	Enlarged gastric folds	12 (22%)
	Sticky tenacious mucus	18 (32.7%)
	Xanthoma	3 (5.4%)
*H. pylori*-negative gastric mucosa (*n* = 27)	Fundic gland polyp	4 (14.8%)
	The regular arrangement of collecting venules	25 (92.6%)
	Diffuse redness	14 (51.8%)
	Gastric ulcer	3 (11.1%)
	Normal	9 (33.3%)

This table illustrates the characteristics of the involved patients regarding their age, gender, residence, level of education, and general clinical presentation. Endoscopic features are also listed in relation to gastric mucosa *H. pylori* positive and negative groups

**Fig. 1:**
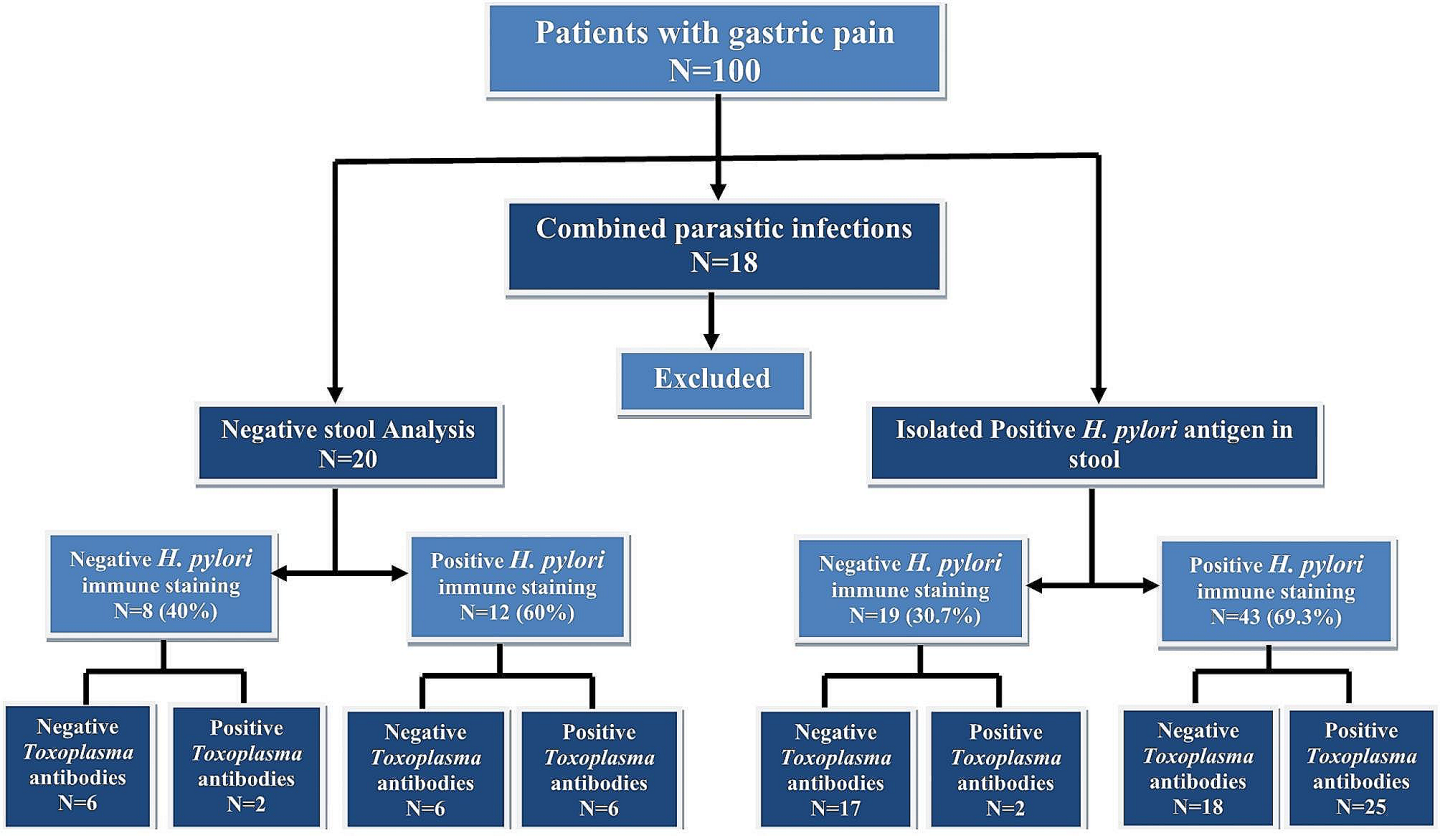
Flow chart of the laboratory results of the studied groups demonstrating excluded cases, different methods used to diagnose H. pylori and study design

Of the 82 patients, 62 were positive for *H. pylori* antigen in stool, and 20 were negative. Histopathological analysis revealed that only 55 patients had *H. pylori*-positive gastric mucosa, and 27 had *H. pylori*-negative mucosa. *Toxoplasma* screening was done for all the patients; 35 patients had positive *Toxoplasma* antibodies (Fig. 1).

**Fig. 2:**
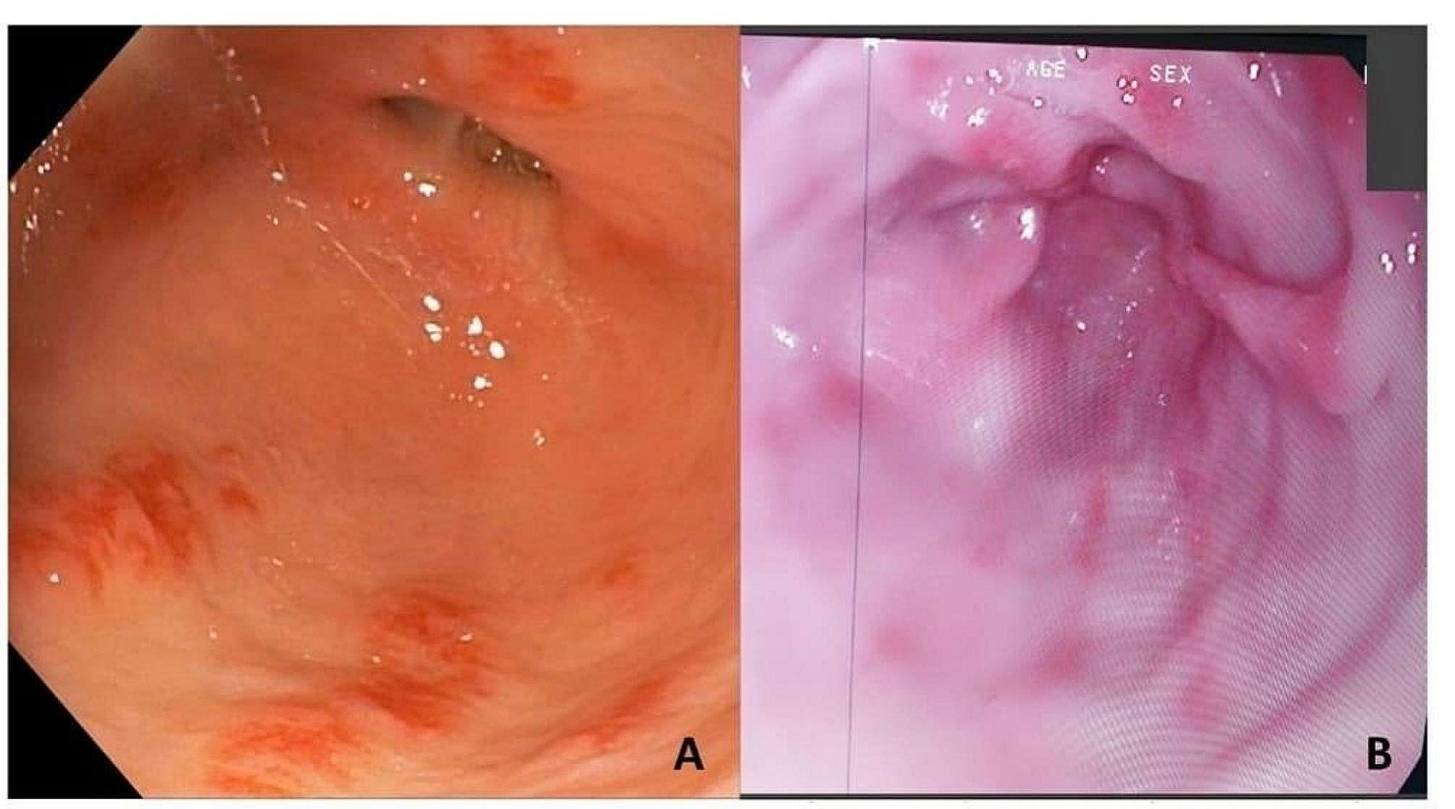
Examples of endoscopic findings in patients with *H pylori* infection, where Fig. 2A shows patchy antral gastritis and erythematous mottling, and Fig. 2B demonstrates moderate antral gastritis and erythematous mottling

Diffuse redness, antral nodularity, spotty fundal hemorrhage, enlarged gastric folds, sticky tenacious mucus and xanthoma were the prominent endoscopic findings in patients with *H. pylori*-associated chronic gastritis. The fundic gland polyp, regular arrangement of collecting venules, raised erosion, and hematin spots were the commonest findings in patients with *H. pylori*-negative gastric mucosa (Table 2; Fig. 2).

According to the previous screenings, we further divided the 82 patients with abdominal pain into 3 groups; the first group included 27 patients negative for both *H. pylori* and *Toxoplasma* gondii infection, 19 (70.4%) of them were males, and 8 (29.6%) were females, their mean age was 33.74 ± 5.9 years. The second group included 24 patients with isolated *H. pylori* infection, which included 17 males (70.8%) and 7 females (29.2%), with a mean age of 34.5 ± 6.2 years. The third group included 20 males (64.5%) and 11 females (35.5%) positive for the two pathogens; their mean age was 33.68 ± 8 years. The gender and age distribution were comparable between the 3 groups with no statistically significant difference (P = < 0.05).

The patients who were negative for both *H. pylori* and *Toxoplasma* infection showed significantly better histopathological criteria than patients with either single or combined *H. pylori* infection. All the patients with negative screenings had no activity, atrophy, intestinal metaplasia, or atopy, and just 14.8% had moderate to severe inflammation (*P* < 0.05).

**Fig. 3:**
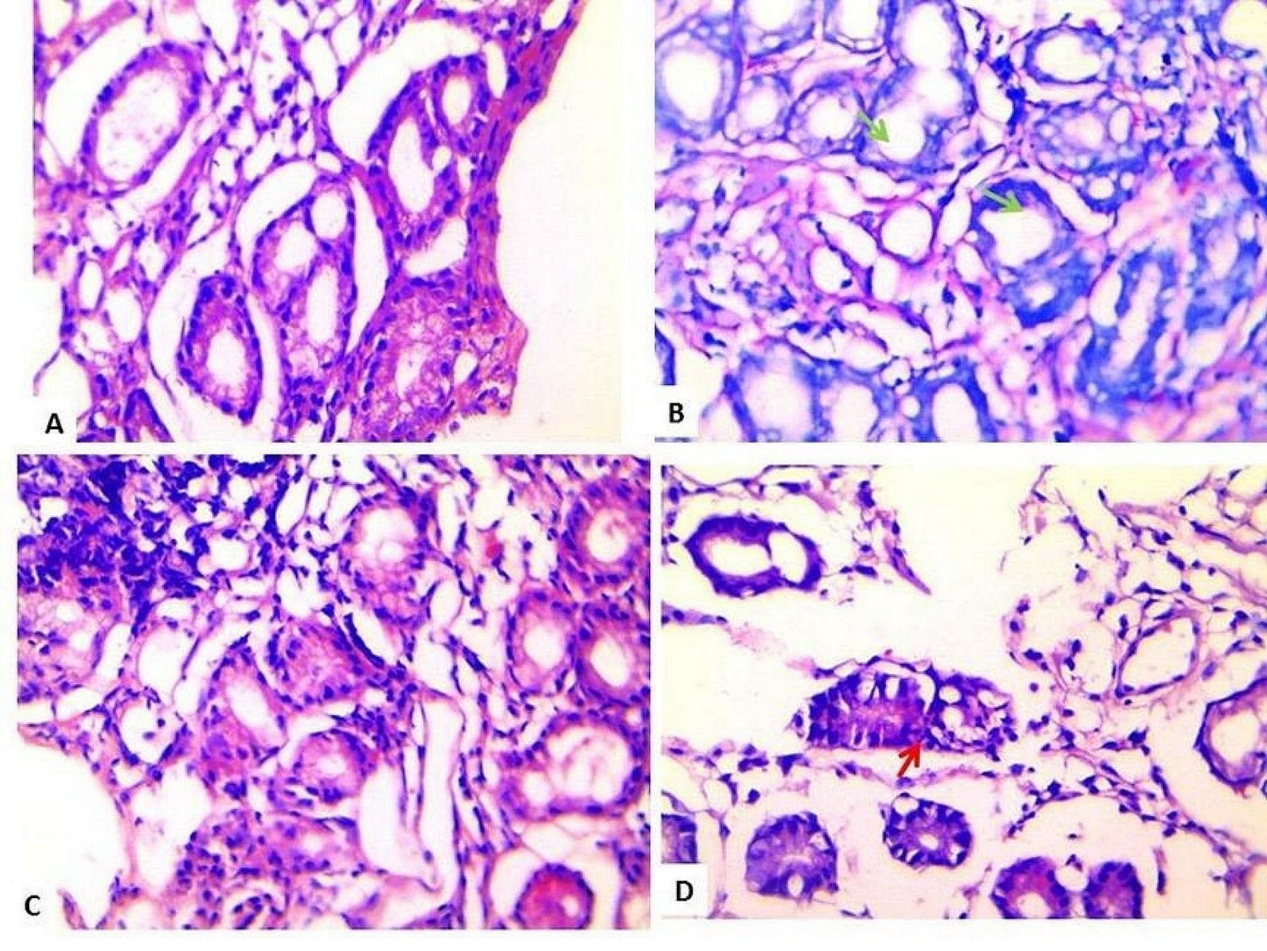
Histopathological examination of gastric biopsy stained with Giemsa and H & E. (A) Gastric biopsy showing mucosal gastric glands containing scattered *H. pylori* bacilli and the intervening lamina propria showed moderate infiltration by acute and chronic inflammatory cells (H & E x400). (B) *H pylori* bacilli carpeting lumen of gastric glands [green arrow] (Geimsa x 400). (C) Severe activity in case of *H. pylori* associated chronic gastritis associated with *T. gondii* infection (H&E x400) (D) *H. pylori* -Severely active gastritis with neutrophils [red arrow] attacking mucosal associated with *T. gondii* infection (H & E x 400)

On comparing patients with isolated *H. pylori* versus combined *H. pylori* and *Toxoplasma* gondii infection, we found that patients with combined *H. pylori* and *Toxoplasma* gondii infection had significantly marked inflammation than patients with isolated infection (*P* < 0.05). At the same time, there was no statistically significant difference in the degree of activity and occurrence of atrophy, intestinal metaplasia, or atopy (*P* > 0.05) (Table 3; Fig. 3). There was no significant correlation on correlating the *Toxoplasma* antibodies titer with the chronic inflammation stage (*r* = 0.167, *P* = 0.395) and degree of activity (*r* = 0.103, *P* = 0.600).

**Table 3:** Comparison between the histopathological findings in single *H. pylori* infection and combined infection with *T. gondii.* Different histopathological features induced by H. pylori and Toxoplasma coinfection

		Group2		P-value
		Single H. pylori infection *n* = 24 N (%)	Combined H. pylori + Toxoplasma infection *n* = 31 N (%)	
Chronic inflammation	absent or mild moderate or severe	12 (50.0%) 12 (50.0%)	6 (19.4%) 25 (80.6%)	0.016٭
Activity	absent or mild moderate or severe	13 (54.2%) 11 (45.8%)	17 (54.8%) 14 (45.2%)	0.960
Atrophy	Absent Focal	22 (91.7%) 2 (8.3%)	29 (93.5%) 2 (6.5%)	1
Intestinal metaplasia	Absent Present	20 (83.3%) 4 (16.7%)	29 (93.5%) 2 (6.5%)	0.387
Atypia	Absent Mild atypia	22 (91.7%) 2 (8.3%)	31 (100%) 0 (0%)	0.186

Different histopathological changes in response to either single infection with *H. pylori* or concurrent infection with toxoplasmosis were evaluated where significant differences were reported concerning chronic inflammation.*Significant differences

**Fig. 4:**
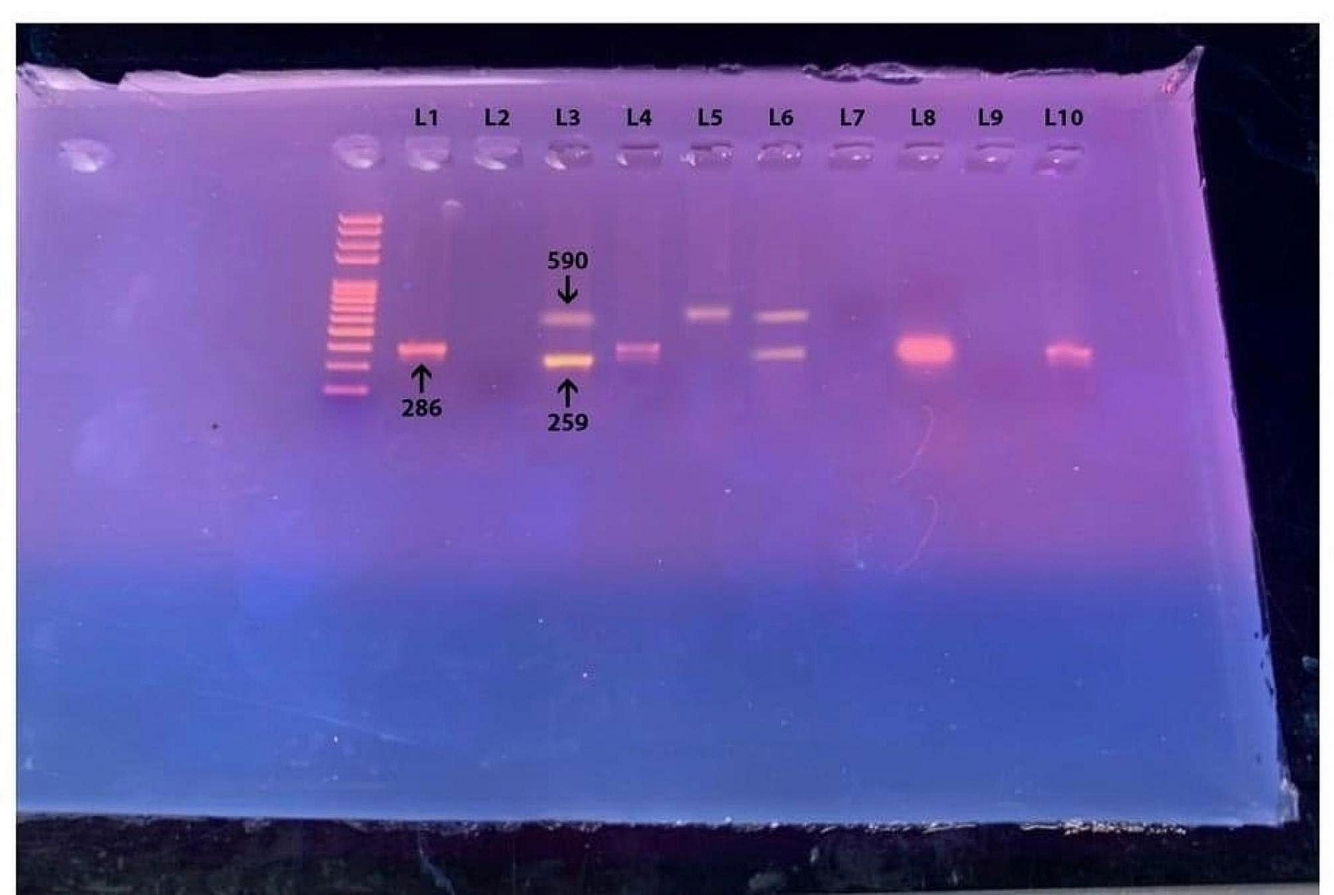
PCR of CagA and VacA genotypes: Lanes 1, 3, 4, 6, 10 Vac A-positive and lane 3, 5, 6 Cag A-positive and lane 2, 7, 9 are negative and L1 is Vac A-positive control while L2 is negative control

Further screening for the virulent strains of *H. pylori* was done for patients with positive immune staining. Out of the 55 patients with positive immune staining, 37 patients had isolated *Vac As1* variants, 11 had combined *Vac As1* and *Cag A* variants, and 7 had combined *Vac As1*, *Cag A* and Vac *As2* variants. Patients with the combined 2 or 3 variances showed significantly deteriorated histopathological features than patients with a single *Vac As1* variant (*P* < 0.05) (Table 4; Fig. 4).

**Table 4:** Histopathological findings in the different genotypes of virulence strains of *H. pylori*

		H.pylori gene			P-value
		Vac As1 *N* = 37	Vac As1+ Cag A *N* = 11	Vac As1+ Cag A + VacAs2 *N* = 7	
Chronic inflammation	absent or mild moderate or severe	18 (48.6%) 19 (51.4%)	0 (0%) 11 (100%)	0 (0%) 7 (100%)	0.001٭
Activity	absent or mild moderate or severe	29 (78.4%) 8 (21.6%)	1 (9.1%) 10 (90.9%)	0 (0%) 7 (100%)	< 0.0001٭
Atrophy	Absent Focal	37 (100%) 0 (0%)	7 (63.6%) 4 (36.4%)	7 (100%) 0 (0%)	< 0.0001٭
Intestinal metaplasia	Absent Present	35 (94.6%) 2 (5.4%)	11 (100.0%) 0 (0.0%)	3 (42.9%) 4 (57.1%)	< 0.0001٭
Atypia	Absent Mild atypia	37 (100.0%) 0 (0.0%)	9 (81.8%) 2 (18.2%)	7 (100.0%) 0 (0.0%)	0.016٭

Histopathological changes in response to different *H. pylori* genes were reported in the above table. Significant differences were documented in different types of genes concerning chronic inflammation, activity, atrophy, intestinal metaplasia, and atypia of gastric mucosa.

*Significant differences

## Discussion

The global prevalent *H. pylori* infection is known to have the potential rise to various complications in the gastroduodenal region, including cancer development [2]. However, its successful eradication is predicted to decrease the occurrence of gastric cancer significantly (GC) [20].

The present study revealed a substantial prevalence rate of 75.6% and 67% among symptomatic patients who sought medical care at the Outpatient Clinics of the Gastroenterology unit at NLI using *H. pylori* antigen in stools and histopathological examination as diagnostic procedures, respectively. These findings were close to a recent study by Kishk et al. [22] and reports from Africa, which exhibited the highest prevalence of H pylori infection, with a rate of 70.1%. In contrast, Oceania demonstrated the lowest prevalence, with a rate of 24.4%. The prevalence also exhibited significant variation among different countries, ranging from a minimum of 18.9% in Switzerland to a maximum of 87.7% in Nigeria [19].

The variations in infection rates were attributed to differences in research methodology, participant diversity and inclusion criteria, number of participants, and approaches used for detecting *H. pylori* [15].

In the present study, the assessment of infection with *H. pylori* was conducted using different diagnostic procedures for symptomatic patients. Our study revealed false positive results detected by the SAT exhibited and were negative in gastric biopsies. These results contrasted with a recent study conducted in Uganda, which emphasized the exceptional role of the SAT in detecting *H. pylori* in regions with a high prevalence of the disease Owot et al. [24] and agreed with the results of Zubair et al. [33] who reported the limited sensitivity and diagnostic accuracy of the HPSA test.

The study found that several socio-demographic variables were associated with *H. pylori* infection. These variables included increasing age, residing in rural areas, and lower educational level, which agreed with previously studied risk factors for infection in earlier studies in Egypt and African countries [3, 15]. Abdominal pain and dyspepsia were the presenting symptoms of most cases in the current work, which agreed with many researchers as they contributed to many cases of infection with *H. pylori* [15, 22].

In our study, screening for the virulent strains of *H. pylori* was done for patients with positive immune staining; 37/55 (67%) patients had isolated *Vac As1* variant, 11/55 (20%) patients had combined *Vac As1* and *Cag A* variants and 7 /55 ( 12.7%) patients had combined *Vac As1*, *Cag A* and Vac *As2* variants. Similar results were obtained by Kishk et al. [22], who showed that all strains with the CagA gene were VacA gene positive.

Concerning the genotypes of CagA and VacA, it was observed that the VacA s1 allele, which was the most prevalent, agreed with a prior investigation conducted on patients from Cuba; the presence of the VacA gene was observed in 61.6% of the *H. pylori* strains examined, with this gene being identified as the predominant virulence factor in most of the strains [13]. Nevertheless, the findings of this study opposed the previous research conducted by Kishk et al. [22] [12], where they observed that the VacA+/CagA − genotype s1 had the lowest prevalence. Conversely, a separate study conducted in Algeria revealed the presence of the CagA gene in 58% of the examined patients [5].

Interestingly, CagA + was not found in our study as a single genotype. It was rather linked to VacA s1 in (11/55) or to Vac *As2* alle in (7/55) patients, and this was consistent with [22]. Additionally, the lower percentage of the VacA s2 genotype is considered a less virulent form as compared with the acutely damaging VacA s1, as stated by Falsafi et al. [12]. This study investigated the association between the VacA and CagA genotypes and clinical outcomes, as endoscopic results showed. Patients with the combined 2 or 3 variances showed significantly deteriorated histopathological features than patients with a single *Vac As1* variant (*P* < 0.05).

The significant impact of association of *H. pylori* and *T. gondii* on gastric inflammation severity was reported in this study, which is documented for the first time based on research on different databases.

The potential correlation between *H. pylori* and *T. gondii* was investigated in the context of gastritis and the development of peptic ulcers. The observed correlation can be attributed to the fecal-oral route, a frequently seen infection pathway for both pathogens. The elevated rates of *H. pylori* discovery in feline fecal samples have prompted consideration of potential zoonotic transmission of these infections [6, 16].

In earlier studies, the co-infection of *H. pylori* with either Ascaris lumbricoides or *T. gondii* resulted in modified *H. pylori* gastritis in mouse experimental models [10]. The present study’s findings indicate that among a cohort of 31 individuals who were simultaneously infected with both *H. pylori* and *T. gondii*, a substantial majority (80.6%) experienced gastritis of moderate to severe intensity. The results were in line with Ghazy et al. [16].

This report presents novel findings regarding the impact of toxoplasmosis on the severity of gastritis in humans. Specifically, it is the first documented record to investigate and compare patients with isolated *H. pylori* infection and those with combined *H. pylori* and *T. gondii* infection. The results indicated that patients with combined infections exhibited significantly more advanced inflammation stages than patients with isolated infections (*P* < 0.05). However, when examining the relationship between the titer of *Toxoplasma* antibodies and the stage of chronic inflammation (*r* = 0.167, *P* = 0.395) as well as the degree of activity (*r* = 0.103, *P* = 0.600), no statistically significant association was seen for either variable. The findings of this study were incongruent with the research conducted by Ghazy et al. [16] since their study demonstrated a positive correlation between the severity of gastritis cases and elevated levels of serum anti-*Toxoplasma* IgG.

## Conclusion and recommendations

Prevalence of chronic *T. gondii* infection in individuals with concomitant *H. pylori* infection. The results revealed an elevated prevalence of latent *Toxoplasma* infection among patients infected with *H. pylori*. Furthermore, a notable association was observed about heightened gastrointestinal severity. Furthermore, the predominant genotype observed in our examined population was *Vac As1*. However, there were notable variations in genotype combinations associated with significantly more severe histopathological manifestations than individuals with a single *Vac As1* variant (*P* < 0.05). Advanced molecular techniques have emerged as reliable means for identifying virulent strains of *H. pylori* due to their enhanced sensitivity and specificity. Future studies are required to examine the mechanism lying behind this association.

## Data Availability

The datasets present in the current investigation can be upon a reasonable request.Contact prof. Marwa Ahmed Gouda Email:

## Abbreviations

T.gondii: *Toxoplasma gondii*
*H. pylori*: *Helicobacter pylori*
*Cag*A: The cytotoxin-associated gene A
PUD: peptic ulcer disease
GC: gastric cancer
*Vac*A: Vacuolatingcytotoxin A gene
*Hp*SA: H. pylori stool antigen
EIA: enzyme immunoassay
